# Time-dependent activation of MAPK/Erk1/2 and Akt/GSK3 cascades: modulation by agomelatine

**DOI:** 10.1186/s12868-014-0119-1

**Published:** 2014-10-21

**Authors:** Laura Musazzi, Mara Seguini, Alessandra Mallei, Giulia Treccani, Mariagrazia Pelizzari, Paolo Tornese, Giorgio Racagni, Daniela Tardito

**Affiliations:** Laboratory of Neuropsychopharmacology and Functional Neurogenomics, Dipartimento di Scienze Farmacologiche e Biomolecolari and Center of Excellence on Neurodegenerative Diseases (CEND), Università degli Studi di Milano, Via Balzaretti 9, Milano, 20133 Italy; Istituto di Ricovero e Cura a Carattere Scientifico Centro S. Giovanni di Dio-Fatebenefratelli, Brescia, Italy

**Keywords:** Antidepressant, Agomelatine, Intracellular signalling, Neuroplasticity, Time-dependent regulation

## Abstract

**Background:**

The novel antidepressant agomelatine, a melatonergic MT_1_/MT_2_ agonist combined with 5-HT_2c_ serotonin antagonist properties, showed antidepressant action in preclinical and clinical studies. There is a general agreement that the therapeutic action of antidepressants needs the activation of slow-onset adaptations in downstream signalling pathways finally regulating neuroplasticity. In the last several years, particular attention was given to cAMP-responsive element binding protein (CREB)-related pathways, since it was shown that chronic antidepressants increase CREB phosphorylation and transcriptional activity, through the activation of calcium/calmodulin-dependent (CaM) and mitogen activated protein kinase cascades (MAPK/Erk1/2).

Aim of this work was to analyse possible effects of chronic agomelatine on time-dependent changes of different intracellular signalling pathways in hippocampus and prefrontal/frontal cortex of male rats. To this end, measurements were performed 1 h or 16 h after the last agomelatine or vehicle injection.

**Results:**

We have found that in naïve rats chronic agomelatine, contrary to traditional antidepressants, did not increase CREB phosphorylation, but modulates the time-dependent regulation of MAPK/Erk1/2 and Akt/glycogen synthase kinase-3 (GSK-3) pathways.

**Conclusion:**

Our results suggest that the intracellular molecular mechanisms modulated by chronic agomelatine may be partly different from those of traditional antidepressants and involve the time-dependent regulation of MAPK/Erk1/2 and Akt/GSK-3 signalling pathways. This could exert a role in the antidepressant efficacy of the drug.

## Background

Regulation of gene expression represents a major component in the mechanism of action of antidepressants [1-3]. Converging evidence shows that a common target of chronic antidepressants is a positive modulation of cAMP-responsive element binding protein (CREB), a transcription factor that regulates the expression of several genes involved in the control of neuroplasticity, circadian rhythms, cell survival and cognition [4-9]. CREB transcriptional activity is regulated by phosphorylation at Ser133, which is induced by multiple signalling cascades. In particular, it was shown that the calcium/calmodulin (CaM)-dependent and the mitogen activated protein (MAP) kinase cascades have a crucial role in the activation of CREB consequent to chronic antidepressant treatments [10-12].

Moreover, recent preclinical biochemical and behavioural evidence suggested an involvement of the Akt/glycogen synthase kinase-3 (GSK-3) signalling, regulating gene expression through the activation of CREB and other transcription factors, both in the modulation of behaviour and in the mechanism of action of psychoactive drugs [13-15]. GSK-3 is a Ser/Thr kinase regulated predominantly in an inhibitory manner through phosphorylation at N-terminal serine residues (Ser^21^ in GSK-3α and Ser^9^ in GSK-3β) by several protein kinases, including Akt [16].

GSK-3β has been linked to bipolar disorder, depression, and schizophrenia [17-19]. It was recently shown that selective serotonin reuptake inhibitors (SSRI) and other 5-HT-related antidepressants (MAO, tricyclic antidepressants) inhibit GSK-3β in many brain regions [14,20,21] and GSK-3 inhibitors showed antidepressant-like action in behavioural tests [22,23]. GSK-3 was also reported to be required for the rapid antidepressant actions of ketamine [24].

The antidepressant agomelatine is an agonist of melatonergic MT_1_/MT_2_ receptors and an antagonist of 5-HT_2c_ receptors. The unique and novel pharmacological profile of agomelatine has been found to be effective in the treatment of depressive symptoms, with a rapid stabilisation of circadian rhythms and a favourable tolerability profile. Efficacy of agomelatine in behavioral tests has been previously reported in different animal models [25-29]. Thus far, little is known about the signalling pathways affected by agomelatine downstream of receptor modulation. This is especially interesting with regard to the short half-life (1–2 h) of agomelatine, its mechanism of action and the signalling pathways responsible for the changes in gene expression and therapeutic effect.

In this study, we verified whether agomelatine modulates the time-dependent activity of some major signalling pathways known to be regulated by traditional antidepressants. In particular, the expression and activation (phosphorylation) of CREB, αCaM kinase II, MAP kinase/Erk1/2, Akt and GSK-3β were investigated at both nuclear and cytoplasmic level of hippocampus (HPC) and prefrontal and frontal cortex (PFC/FC) of rats treated for three weeks with agomelatine or vehicle and sacrificed 1 h (6 p.m.) or 16 h (9 a.m.) after the last drug administration.

We found that agomelatine, rather than potentiating CREB-related signalling as traditional antidepressants, was able to partly modulate the time-dependent activation of the MAP/Erk1/2 and Akt/GSK cascades, suggesting that its antidepressant properties might be related with a fine-tuning of the time-dependent oscillations in forebrain intracellular signalling.

## Results

### Chronic agomelatine does not activate CREB

**Figure 1:**
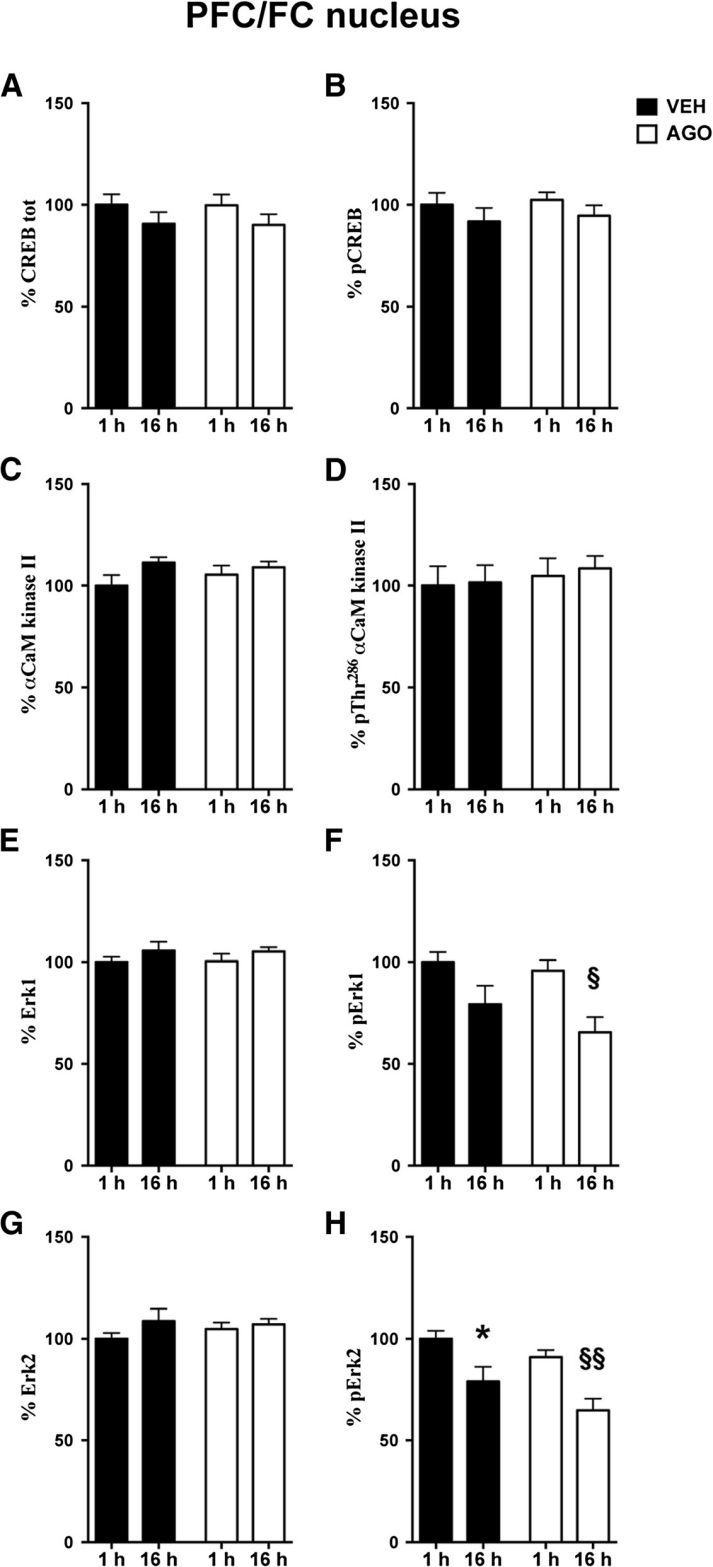
**Time-dependent fluctuation of CREB, αCaM kinase II, MAPK/Erk1/2 in PFC/FC nuclei.** Modulation by chronic agomelatine. Expression levels of CREB **(A)**, αCaM kinase II **(C)**, MAPK/Erk1 **(E)** and MAPK/Erk2 **(G)** and phosphorylation levels of CREB at Ser^133^ **(B)**, αCaM kinase II at Thr^286^ **(D)**, MAPK/Erk1 at Thr^202/204^ **(F)** and MAPK/Erk2 at Thr^202/204^ **(H)** in nuclear fraction of PFC/FC from rats chronically treated with vehicle (VEH, black bar) or agomelatine (AGO, white bar) and sacrificed 1 or 16 h after the last injection. Data are expressed as % intensity units/mm^2^ (mean ± S.E.M.). Bonferroni post-hoc test (following 2-way ANOVA): *p < 0.05 VEH treated rats sacrificed 16 h after last injection vs. VEH treated rats sacrificed 1 h after last injection; § p < 0.05 AGO 16 h after last injection vs. AGO 1 h after last injection; §§p < 0.01 AGO 16 h after last injection vs. AGO 1 h after last injection (n =8 rats/group).

**Figure 2:**
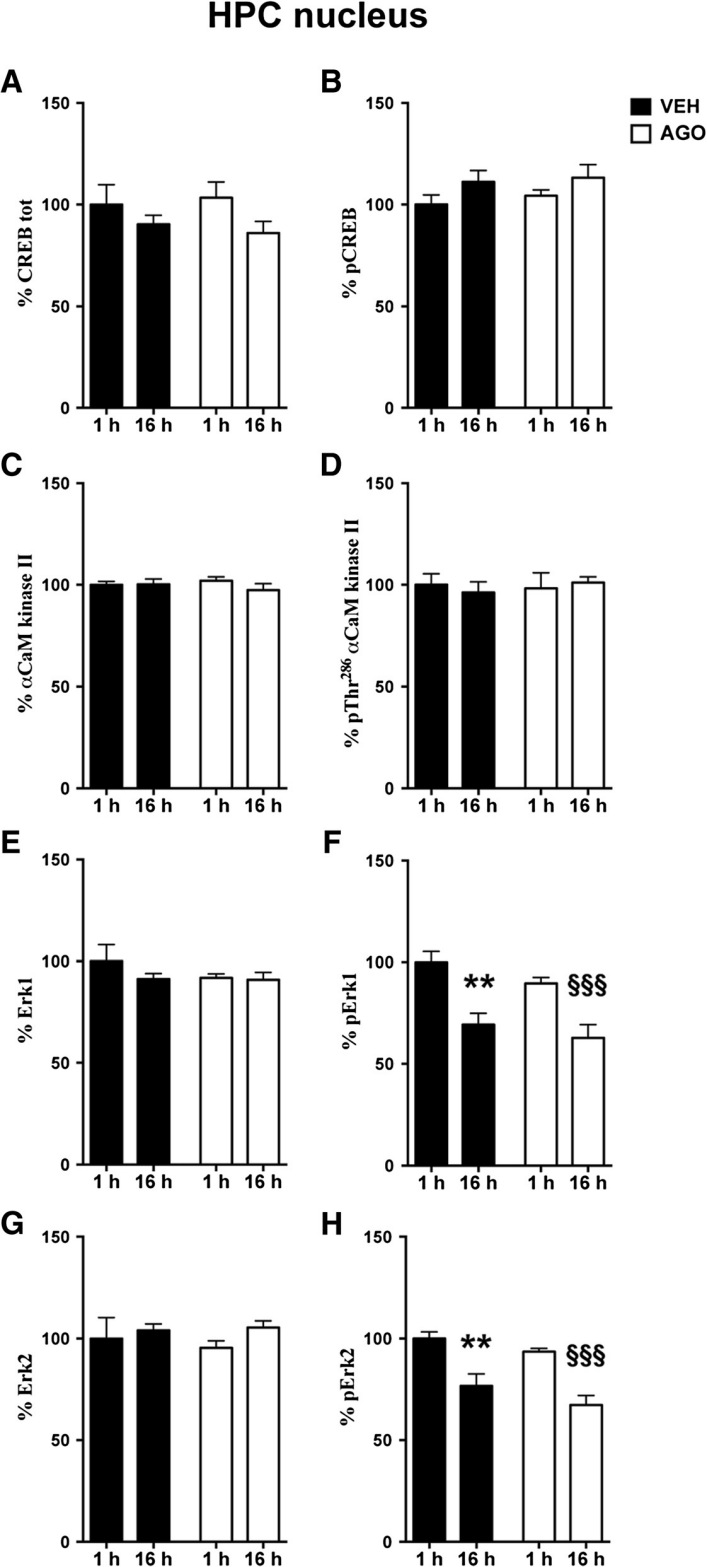
**Time-dependent fluctuation of CREB, αCaM kinase II, MAPK/Erk1/2 in HPC nuclei. Modulation by chronic agomelatine.** Expression levels of CREB **(A)**, αCaM kinase II **(C)**, MAPK/Erk1 **(E)** and MAPK/Erk2 **(G)** and phosphorylation levels of CREB at Ser^133^ **(B)**, αCaM kinase II at Thr^286^ **(D)**, MAPK/Erk1 at Thr^202/204^ **(F)** and MAPK/Erk2 at Thr^202/204^ **(H)** in nuclear fraction of HPC from rats chronically treated with vehicle (VEH, black bar) or agomelatine (AGO, white bar) and sacrificed 1 or 16 h after the last injection. Data expressed as above. Statistics as above. **p < 0.01 VEH 16 h after last injection vs. VEH 1 h after last injection; §§§p < 0.001 AGO 16 h after last injection vs. AGO 1 h after last injection (n =8 rats/group).

In order to dissect the effect of chronic agomelatine on the signalling pathways regulating CREB activity and its putative time-dependent modulation, rats were chronically treated with vehicle or agomelatine (once daily for 21 days) at 5 p.m. and sacrificed after 1 h (6 p.m.) or 16 hours (9 a.m). CREB expression and activation (phosphorylation at Ser133) were measured in PFC/FC and HPC nuclear fractions. The two-way ANOVA highlighted no time-related changes in CREB expression or phosphorylation levels between 1 h or 16 h post-treatment and, interestingly, differently from traditional antidepressants [11], chronic agomelatine did not induce any significant changes in CREB expression or phosphorylation (Figure 1A,B for PFC/FC and Figure 2A,B for HPC).

### Chronic agomelatine modulates time-dependent changes of MAPK/Erk1/2 activation in frontal/prefrontal cortex but not in hippocampus

Different intracellular signalling cascades, including CaM kinases and MAP kinases, are known to modulate CREB phosphorylation in the action of antidepressants [10-12]. To investigate whether the lack of CREB activation after chronic agomelatine was dependent on a lack of activation or a dysregulation of intracellular signalling cascades regulating CREB phosphorylation, we assessed the expression and phosphorylation levels of αCaM Kinase II and MAPK/Erk1/2, in PFC/FC and HPC nuclear fractions following agomelatine treatment.

Interestingly, in both PFC/FC and HPC, we found that expression and phosphorylation levels of αCaMKII were unmodified by both time and agomelatine (Figure 1C, D for PFC/FC; Figure 2C, D for HPC). On the contrary, the activation of MAPK/Erk1/2, through phosphorylation at Thr^202^/Tyr^204^ residues, was found to be significantly lower in animals sacrificed in the morning (9 a.m*.*), than in the evening (6 p.m.), in nuclei from both PFC/FC (2-way ANOVA, significant effect of time, pERK1: F(1,26) = 12.88, p < 0.01; pERK2: F(1,26) = 18.55, p < 0.001) and HPC (2-way ANOVA: significant effect of time, pERK1: F(1,26) = 28.50, p < 0.0001; pERK2: F(1,26) = 31.72, p < 0.0001) (Figure 1F,H for PFC/FC; Figure 2F,H for HPC).

Moreover, in PFC/FC, the 2-Way ANOVA highlighted a significant effect also of treatment for pErk2 (F(1,26) = 4.49, p < 0.05) (Figure 1H), and the reduction of pErk1 levels at 16 h was significant only for treated rats (Bonferroni post-hoc test: AGO 16 h vs. 1 h −30.31% p < 0.05) (Figure 1F), thus suggesting that agomelatine reduces Erk2 activation and potentiates the time-dependent modulation of pErk1 in PFC/FC. On the contrary, the time-dependent changes in MAPK/Erk1/2 phosphorylation were similar in HPC of treated and untreated animals (Figure 2F,H).

### Modulation of time-dependent activation of the Akt/GSK-3 pathway by chronic agomelatine in frontal/prefrontal cortex and hippocampus

**Figure 3:**
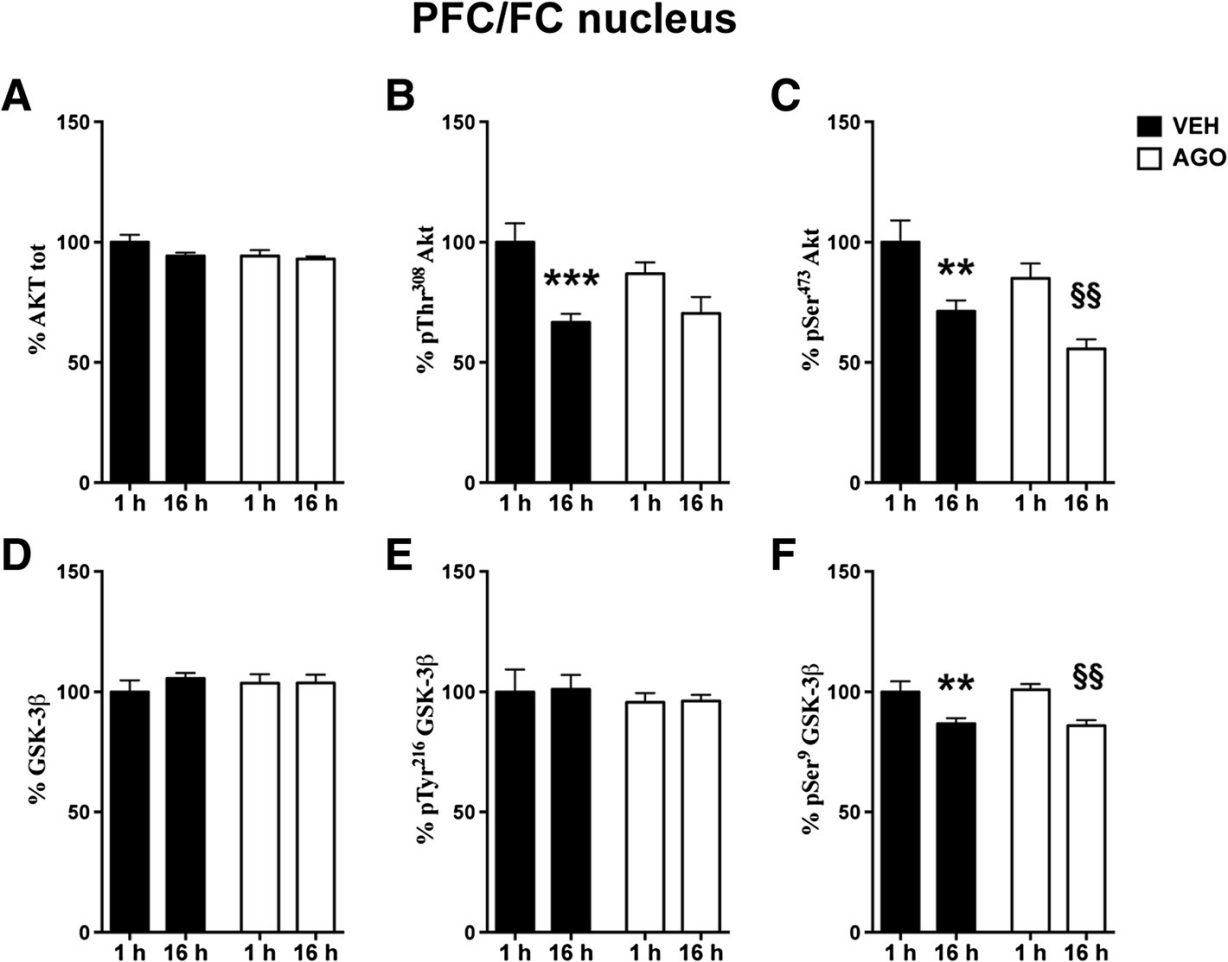
**Time-dependent fluctuation of Akt/GSK-3 signalling pathway in PFC/FC nuclear fraction. Modulation by chronic agomelatine.** Expression levels of Akt **(A)** and phosphorylation levels of Akt at Thr^308^ **(B)** and at Ser^473^ **(C)**; expression levels of GSK-3 β **(D)**; phosphorylation levels of GSK-3 β at Tyr^216^ **(E)** and at Ser^9^ **(F)** in nuclear fraction of PFC/FC from rats chronically treated with vehicle (VEH, black bar) or agomelatine (AGO, white bar) and sacrificed 1 or 16 h after the last injection. Data expressed as above. Statistics as above. **p < 0.01, ***p < 0.001 VEH 16 h after last injection vs. VEH 1 h after last injection; §§p <0.01 AGO 16 h after last injection vs. AGO 1 h after last injection (n =8 rats/group).

**Figure 4:**
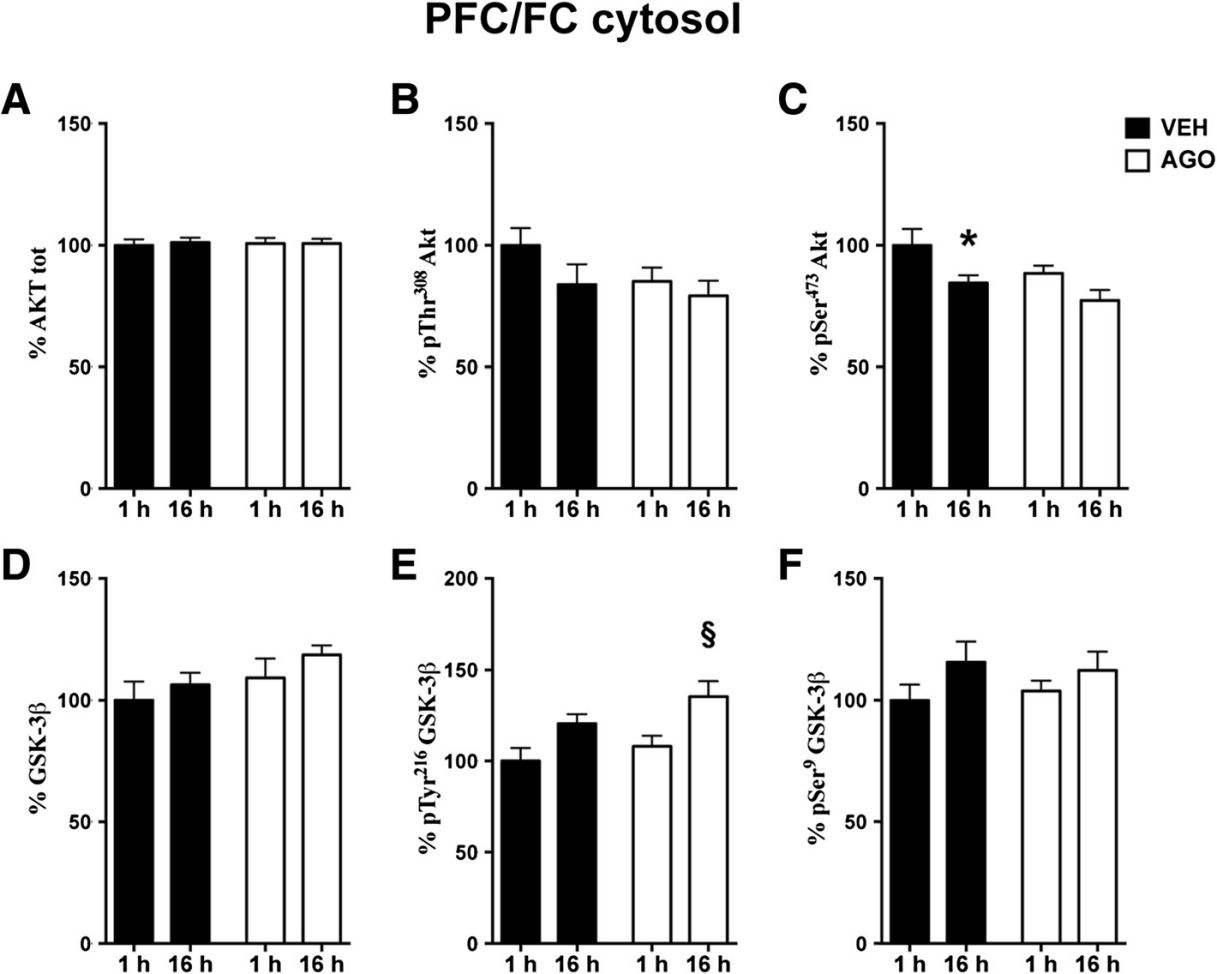
**Time-dependent fluctuation of Akt/GSK-3 signalling pathway in PFC/FC cytosolic fraction and modulation by chronic agomelatine.** Expression levels of Akt **(A)** and phosphorylation levels of Akt at Thr^308^ **(B)** and at Ser^473^ **(C)**; expression levels of GSK-3 β **(D)**; phosphorylation levels of GSK-3 β at Tyr^216^ **(E)** and at Ser^9^ **(F)** in cytosolic fraction of PFC/FC from rats chronically treated with vehicle (VEH, black bar) or agomelatine (AGO, white bar) and sacrificed 1 or 16 h after the last injection. Data expressed as above. Statistics as above. *p < 0.05 VEH 16 h after last injection vs. VEH 1 h after last injection; §p < 0.05 AGO 16 h after last injection vs. AGO 1 h after last injection (n =8 rats/group).

**Figure 5:**
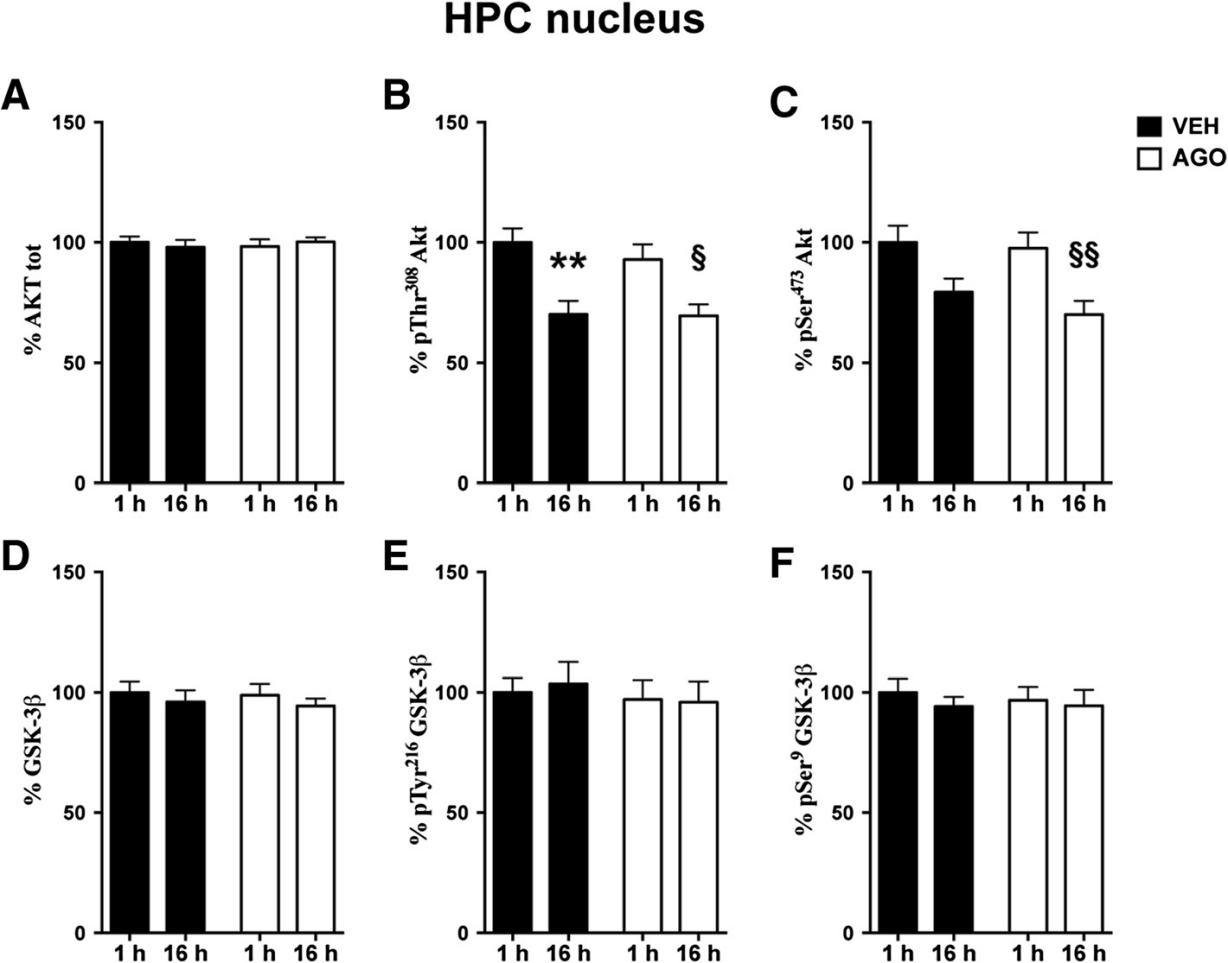
**Time-dependent fluctuation of Akt/GSK-3 signalling pathway in HPC nuclear fraction and modulation by chronic agomelatine.** Expression levels of Akt **(A)** and phosphorylation levels of Akt at Thr^308^ **(B)** and at Ser^473^ **(C)**; expression levels of GSK-3 β **(D)**; phosphorylation levels of GSK-3 β at Tyr^216^ **(E)** and at Ser^9^ **(F)** in nuclear fraction of HPC from rats chronically treated with vehicle (VEH, black bar) or agomelatine (AGO, white bar) and sacrificed 1 or 16 h after the last injection. Data expressed as above. Statistics as above. **p < 0.01 VEH 16 h after last injection vs. VEH 1 h after last injection; §p < 0.05, §§p < 0.01 AGO 16 h after last injection vs. AGO 1 h after last injection (n =8 rats/group).

**Figure 6:**
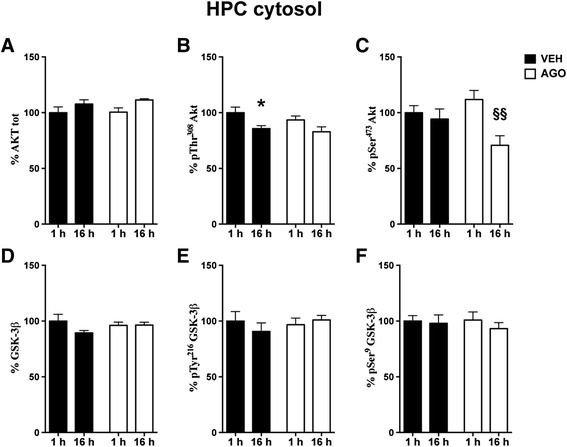
**Time-dependent fluctuation of Akt/GSK-3 signalling pathway in HPC cytosolic fraction and modulation by chronic agomelatine.** Expression levels of Akt **(A)** and phosphorylation levels of Akt at Thr^308^ **(B)** and at Ser^473^ **(C)**; expression levels of GSK-3 β **(D)**; phosphorylation levels of GSK-3 β at Tyr^216^ **(E)** and at Ser^9^ **(F)** in cytosolic fraction of HPC from rats chronically treated with vehicle (VEH, black bar) or agomelatine (AGO, white bar) and sacrificed 1 or 16 h after the last injection. Data expressed as above. Statistics as above. *p < 0.05 VEH 16 h after last injection vs. VEH 1 h after last injection; §§p < 0.01 AGO 16h after last injection vs. AGO 1h after last injection (n =8 rats/group).

To assess whether agomelatine can modulate the Akt/GSK-3 signalling pathway, we measured total expression and phosphorylation levels of Akt and GSK-3β in nuclear and cytosolic fractions from PFC/FC and HPC of rats chronically treated with agomelatine and sacrificed at 6 p.m. or 9 a.m*.* As described in details below, although the expression levels of Akt and GSK-3β were unmodified by chronic agomelatine, the phosphorylation levels of the kinases were partly and selectively regulated by the drug, depending on the brain area and subcellular fraction analysed (Figures 3, 4, 5, 6). Interestingly, in some cases, chronic agomelatine was able to modulate the activation of Akt/GSK-3 phosphorylation, as shown below.

In nuclear fraction from PFC/FC, the 2-way ANOVA showed a significant effect of time on Akt phosphorylation at Thr^308^, which is decreased in rats sacrificed in the morning (F(1,28) = 17.43 p < 0.001) (Figure 3). Interestingly, post-hoc test was significant only for vehicle treated rats (Bonferroni post-hoc test: VEH 16 h vs. 1 h −33.40% p < 0.001) (Figure 3B), suggesting that agomelatine dampens the time-dependent modulation of phospho-Thr^308^ Akt. Instead, 2-way ANOVA reported a significant effect of both time and treatment for phospho-Ser^473^ Akt, whose levels were decreased by agomelatine and were significantly lower in both groups of animals at 16 h vs 1 h (2-way ANOVA: time F(1,28) = 21.54 p < 0.0001, treatment F(1,28) = 5.96 p < 0.05; Bonferroni post-hoc test: VEH 16 h vs. 1 h −28.81% p < 0.01, AGO 16 h vs. 1 h −29.39% p < 0.01) (Figure 3C).

A significant effect of time was also found in nuclear fraction from PFC/FC for phospho-Ser^9^ GSK-3β, which levels were lower in both agomelatine and vehicle treated rats sacrificed in the morning (2-way ANOVA: time F(1,28) = 22.06 p < 0.0001; Bonferroni post-hoc tests: VEH 16 h vs. 1 h −13.23% p < 0.01, AGO 16 h vs. 1 h −15.01% p < 0.01) (Figure 3F).

In cytosol from PFC/FC, a significant effect of time was found on phospho-Ser^473^ Akt levels (2-way ANOVA: time F(1,28) = 8.33 p < 0.01), while post-hoc comparisons were significant only in vehicle treated rats (Bonferroni post-hoc test: VEH 16 h vs. 1 h −15.44% p < 0.05) (Figure 4C). Moreover, 2-way ANOVA showed a significant effect of time on phosphorylation of GSK-3β at Tyr216 (F(1,28) = 12.54 p < 0.01) and the post-hoc test highlighted a significant increase at 16 h selectively in rats chronically treated with agomelatine (Bonferroni post-hoc test: AGO 16 h vs. 1 h +27.33% p < 0.05) (Figure 4E). No significant differences were found in the phosphorylation state of both pTyr^308^ of Akt and pSer^9^ of Gsk-3β in cytosol from PFC/FC (Figure 4B and F, respectively).

A different pattern was found in HPC. Indeed, in the nuclear fraction from HPC, while phospho-Thr308 Akt levels were similarly reduced in the morning in all animals (2-way ANOVA: significant effect of time F(1,28) = 21.92 P < 0.0001; Bonferroni post-hoc tests: VEH 16 h vs. 1 h −29.79% p < 0.01, AGO 16 h vs. 1 h −23.23% p < 0.05) (Figure 5B), the time-dependent reduction of phosphorylation of Akt at Ser^473^ was significant only in rats treated with agomelatine (2-way ANOVA: significant effect of time F(1,28) = 14.89 p < 0.001; Bonferroni post-hoc test: AGO 16 h vs. 1 h −27.47% p < 0.01) (Figure 5C). No differences were found in the phosphorylation state of both pTyr^216^ and Ser^9^ of Gsk-3β in HPC nuclear fraction (Figure 5E and F, respectively).

In the cytosolic fraction from the same area, the 2-way ANOVA highlighted a significant effect of time for phospho-Thr^308^ Akt levels (time F(1,28) = 6.19 P < 0.01) (Figure 6B) and of both time and treatment/time interaction for phospho-Ser^473^ Akt (time F(1,28) = 8.33 P < 0.01; treatment/time interaction F(1,28) = 4.75 P < 0.05) (Figure 6C), thus suggesting that agomelatine exerts a different effect at 16 h vs 1 h. Post-hoc test showed a significant morning decrease of phospho-Thr^308^ Akt levels only in vehicle, but not in agomelatine treated rats (Bonferroni post-hoc test: VEH 1 h vs. 16 h −14.30% p < 0.05) (Figure 6B) and a significant and marked reduction of phospho-Ser^473^ Akt levels at 16 h (Bonferroni post-hoc test: AGO 1 h vs. 16 h −41.12% p < 0.01) (Figure 6C). No difference was found in GSK-3β phosphorylation at both Tyr^216^ and Ser^9^ residues in HPC cytosol (Figure 6E,F).

For clarity, all significant changes in time-dependent regulation of MAPK/Erk1/2 and Akt/GSK-3 pathways, in nuclei and cytosol from PFC/FC and HPC of vehicle or agomelatine treated rats, and relative statistics are resumed in Table 1.

**Table 1:** **Summarizing significant changes in time-dependent regulation of MAPK/Erk1/2 and Akt/GSK-3 pathways, in nuclei and cytosol from PFC/FC and HPC of vehicle (VHE) or agomelatine (AGO) treated rats, sacrificed 1 h or 16 h after last injection**

**Protein (figure number)**	**Brain area**	**Subcellular fraction**	**Variable**	**F statistic**	**2 Way ANOVA P value**	**Significant Bonferroni post-hoc tests**
pERK 1 (Figure 1F)	PFC/FC	nucleus	treatment	F(1,26) = 1.62	P = 0.2147	AGO 16 h vs.
			**time**	**F(1,26) = 12.88**	**P < 0.01**	AGO 1 h **p < 0.05**
			interaction	F(1,26) = 0.46	P = 0.5042	
pERK 2 (Figure 1H)	PFC/FC	nucleus	**treatment**	**F(1,26) = 4.49**	**P < 0.05**	AGO 16 h vs.
			**time**	**F(1,26) = 18.55**	**P < 0.001**	AGO 1 h **p < 0.01**
			interaction	F(1,26) = 0.22	P = 0.6401	
pERK 1 (Figure 2F)	HPC	nucleus	treatment	F(1,26) = 2.45	P = 0.1300	VEH 16 h vs.
			**time**	**F(1,26) = 28.50**	**P < 0.0001**	VEH 1 h **p < 0.01**
			interaction	F(1,26) = 0.13	P = 0.7201	AGO 16 h vs.
						AGO 1 h **p < 0.001**
pERK 2 (Figure 2H)	HPC	nucleus	treatment	F(1,26) = 3.24	P = 0.0833	VEH 16 h vs.
			**time**	**F(1,26) = 31.72**	**P < 0.0001**	VEH 1 h **p < 0.01**
			interaction	F(1,26) = 0.11	P = 0.7476	AGO 16 h vs.
						AGO 1 h **p < 0.001**
pThr^308^ Akt (Figure 3B)	PFC/FC	nucleus	treatment	F(1,28) = 0.61	P = 0.4429	VEH 16 h vs.
			**time**	**F(1,28) = 17.43**	**P < 0.001**	VEH 1 h **p < 0.001**
			interaction	F(1,28) = 2.00	P = 0.1685	
pSer^473^ Akt (Figure 3C)	PFC/FC	nucleus	**treatment**	**F(1,28) = 5.96**	**P < 0.05**	VEH 16 h vs.
			**time**	**F(1,28) = 21.54**	**P < 0.0001**	VEH 1 h **p < 0.01**
			interaction	F(1,28) = 0.00	P = 0.9631	AGO 16 h vs.
						AGO 1 h **p < 0.01**
pSer^9^ GSK-3 β (Figure 3F)	PFC/FC	nucleus	treatment	F(1,28) = 0.00	P = 0.9779	VEH 16 h vs.
			**time**	**F(1,28) = 22.06**	**P < 0.0001**	VEH 1 h **p < 0.01**
			interaction	F(1,28) = 0.09	P = 0.7695	AGO 16 h vs.
						AGO 1 h **p < 0.01**
pSer^473^ Akt (Figure 4C)	PFC/FC	cytosol	treatment	F(1,28) = 4.15	P = 0.0512	VEH 16 h vs.
			**time**	**F(1,28) = 8.33**	**P < 0.01**	VEH 1 h **p < 0.05**
			interaction	F(1,28) = 0.22	P = 0.6429	
pTyr^216^ GSK-3 β (Figure 4E)	PFC/FC	cytosol	treatment	F(1,28) = 2.87	P = 0.1016	AGO 16 h vs.
			**time**	**F(1,28) = 12.54**	**P < 0.05**	AGO 1 h **p < 0.05**
			interaction	F(1,28) = 0.25	P = 0.6183	
pThr^308^ Akt (Figure 5B)	HPC	nucleus	treatment	F(1,28) = 0.46	P = 0.5017	VEH 16 h vs.
			**time**	**F(1,28) = 21.92**	**P < 0.0001**	VEH 1 h **p < 0.01**
			interaction	F(1,28) = 0.34	P = 0.5669	AGO 16 h vs.
						AGO 1 h **p < 0.05**
pSer^473^ Akt (Figure 5C)	HPC	nucleus	treatment	F(1,28) = 0.86	P = 0.3626	AGO 16 h vs.
			**time**	**F(1,28) = 14.89**	**P < 0.001**	AGO 1 h **p < 0.01**
			interaction	F(1,28) = 0.29	P = 0.5928	
Akt tot (Figure 6A)	HPC	cytosol	treatment	F(1,28) = 0.30	P = 0.5863	-
			**time**	**F(1,28) = 6.19**	**P < 0.05**	
			interaction	F(1,28) = 0.18	P = 0.6777	
pThr^308^ Akt (Figure 6B)	HPC	cytosol	treatment	F(1,28) = 1.34	P = 0.2567	VEH 16 h vs.
			**time**	**F(1,28) = 9.61**	**P < 0.01**	VEH 1 h **p < 0.05**
			interaction	F(1,28) = 0.22	P = 0.6447	
pSer^473^ Akt (Figure 6C)	HPC	cytosol	treatment	F(1,28) = 0.52	P = 0.4788	AGO 16 h vs.
			**time**	**F(1,28) = 8.33**	**P < 0.01**	AGO 1 h **p < 0.01**
			**interaction**	**F(1,28) = 4.75**	**P < 0.05**	

Significant effects are highlighted in bold.

## Discussion

The study of the effects of agomelatine on intracellular signalling pathways modulating CREB activation demonstrated that, differently from other antidepressants that activate the transcription factor CREB, mainly through phosphorylation by αCaM kinase II and IV and MAPK/Erk1/2 cascades [11], chronic agomelatine did not act on these molecular mechanisms. Indeed, we did not observe any significant modification in both CREB and αCaM kinase II activation in nuclear fraction from PFC/FC or HPC. Our results are in line with those of Morley-Fletcher et al. [30], who recently showed in the same rat strain, that chronic agomelatine, although able to reverse the reduction of CREB phosphorylation induced by prenatal stress in rats, had no effect on CREB phosphorylation in non-stressed animals.

A number of previous studies have shown that both acute and chronic agomelatine increase the expression of brain-derived neurotrophic factor and of other neuroplastic molecules such as fibroblast growth factor, and activity-regulated cytoskeleton-associated protein [31-35], suggesting that agomelatine could exert neurotrophic and antidepressant effects through the activation of molecular mechanisms partly different from those of other drugs [11,27].

Interestingly, we observed time-dependent changes in the activation of MAPK/Erk1/2 kinases in both PFC/FC and HPC. Indeed, we found significantly lower phosphorylation levels of both kinases in animals sacrificed in the morning as compared to those sacrificed in the evening.

A circadian modulation of MAPK/Erk1/2 activity was recently demonstrated in hippocampus and shown to contribute to memory consolidation [36-38]. Thus, according to our present results, time-related fluctuations in nuclear Erk1/2 activation are present also in PFC/FC, where chronic treatment with agomelatine seems to partly modulate the physiological changes. Further studies are needed to better clarify the circadian nature of these modifications and the potential of agomelatine in the modulation of the MAPK/Erk1/2 signalling pathways.

We also observed a time-dependent modulation of Akt and GSK-3β activation through phosphorylation that was selectively different depending on the brain area and the subcellular fraction analysed. In particular, we found that Akt phosphorylation was lower in PFC/FC and HPC of animals sacrificed in the morning (16 h after the last injection). This down-regulation of the kinase activation is particularly evident in the nuclear fraction, suggesting a possible involvement in the regulation of gene transcription. Moreover, since the Ser^9^ of GSK-3β is phosphorylated by Akt, the reduced phosphorylation of GSK-3β at Ser^9^ observed in nuclei from PFC/FC could be linked to the morning decrease of Akt activity observed in the same fraction. Although previous studies have shown a circadian modulation of Akt and GSK-3 activity, the functional role of these fluctuations is poorly understood. Genetic manipulations, leading to increased Akt activity in neurons of the suprachiasmatic nucleus, lengthen the circadian period of locomotor activity, whereas the reduction of Akt signalling shortens it [39]. Moreover, it was recently demonstrated that both genetic and pharmacological reduction of GSK-3 activity in mouse embryonic fibroblasts have a specific effect on the circadian transcriptional oscillation, showing a phase delay in the transcription of the clock gene mPer2 [40].

Our data suggest that chronic treatment with agomelatine reduces phospho-Akt at Ser^473^ in nuclear fraction of PFC/FC. More interestingly, chronic agomelatine was also shown to partly modulate the time-dependent regulation of Akt and GSK-3β activity. Indeed, agomelatine treatment dampened the morning reduction of Akt phosphorylation at Thr^308^ in nuclear fraction and at Ser^473^ in cytosol of PFC/FC. Moreover, agomelatine induced a significant increase in pTyr^216^ GSK-3β in cytosol from PFC/FC and a decrease of pSer^473^ Akt in nuclei from HPC 16 h after the last drug injection. Finally, in the cytosolic fraction from HPC of rats sacrificed in the morning, agomelatine dampened the phosphorylation levels of Akt at Thr^308^ and induced a significant decrease of pSer^473^ Akt.

Agomelatine treatment for 21 days was previously described to induce an increase in Erk1/2, Akt and GSK-3β phosphorylation after chronic treatment in whole hippocampal extract from HPC of rats sacrificed 16h after last drug administration [31]. The following factors might contribute to explaining the apparent contrasting findings compared to our present results: 1- different rat strains were used, Sprague Dawley rats in our study vs. Wistar rats in the previous study; 2- different antibodies were used; 3- the cellular fractions analysed in the two studies were different (nuclear and cytosol vs total extract). In this regard, it is well known that the subcellular distribution and compartmentalization of ERK-MAPKs between cytosol and nucleus play a key role in the regulation of activity and specificity of the kinases [41,42].

## Conclusion

In summary, we have shown that chronic treatment with the antidepressant agomelatine does not activate CREB and CREB-related signalling in nuclei and cytosol from both HPC and PFC/FC of naïve rats. This is of particular interest because suggests that the antidepressant effect of agomelatine could be mediated by different molecular mechanisms with respect to classical antidepressants. Although further studies are warranted to identify the different pattern of intracellular signalling pathways modulated by agomelatine, this difference might contribute to the therapeutic benefits.

Moreover, although further studies are required to reveal whether MAPK/Erk1/2 and Akt/GSK-3 signalling pathways could play a role in the circadian control in HPC and PFC/FC, the present work has provided clear evidence of a time-dependent modulation of these pathways in both brain areas. Finally, considering the effects of agomelatine on the time-dependent regulation of Erk1/2, Akt and GSK-3β phosphorylation levels, it can be speculated that this could be a target in the antidepressant effect induced by the drug.

## Methods

### Animals

Twenty-four male Sprague–Dawley rats (175–200 g) were purchased from Charles River (Calco, Italy). Animals were kept at constant temperature (22°C) with a regular 12 h light/dark cycle (light-on at 7 a.m.). The rats were housed in stable groups of four compatible individuals, in 800 cm^3^ cages, with sawdust bedding, adequate environmental enrichment and *ad libitum* access to food and water. The wellbeing of all animals was daily monitored.

All animal procedures were conducted according to current regulations for animal experimentation in Italy (Decreto Legislativo 116/1992) and the European Union (European Communities Council Directive 2010/63/EU), were approved by the Italian Ministry of Health (Decreto Legislativo 295/2012-A) and the whole study adheres to the ARRIVE guidelines.

### Drug treatments

Rats were randomly divided in 4 experimental groups (8 animals each): 1- Chronic vehicle (hydroxyethylcelullose 1%, 1 ml/kg, i.p.) sacrificed 1 h after the last injection; 2- Chronic vehicle sacrificed 16 h after the last injection; 3- Chronic agomelatine (40 mg/kg i.p.) sacrificed 1 h after the last injection; 4- Chronic agomelatine sacrificed 16 h after the last injection. Treatments were given once a day for 21 days, at 5.00 p.m. (2 h before the start of the dark cycle, 7 p.m.) The HPC and PFC/FC were quickly dissected on ice and processed for subsequent experiments.

### Preparation of subcellular fractions for signalling studies

Individual HPC and PFC/FC were homogenized in 10 volumes of 0.28 M sucrose buffered at pH 7.4 with Tris, containing 20 mM NaF, 5 mM Na_2_H_2_P_2_O_7_, 1 mM Na_3_VO_4_ (protein phosphatase inhibitors), and 2 μl/ml of protease inhibitor cocktail (Sigma-Aldrich S.r.l., Milan, Italy), using a glass–teflon tissue grinder (clearance 0.25 mm) in order to obtain the total homogenate fraction [11]. The homogenate was further centrifuged (5 min, 1000 g at 4°C) and the resulting pellet (nuclear fraction) was resuspended in lysis buffer (120 mM NaCl, 20 mM HEPES pH 7.4, 0.1 mM EGTA, 0.1 mM DTT, containing 20 mM NaF, 5 mM Na_2_H_2_P_2_O_7_, 1 mM Na_3_VO_4_, and 2 μl/ml of protease inhibitor cocktail). To obtain the cytosolic fraction, the supernatant of the 1000 g centrifugation was ultracentrifuged at 135,000 g for 1 h. Each fraction was immediately stored at −80°C after being obtained. Protein content of subcellular fractions was evaluated by using the BCA Protein Assay (Thermo Fisher Scientific SAS, Illkirch Cedex, France). Purity of subcellular fractions was checked by measuring subcellular distribution of protein markers, as previously shown [43].

### Western blot analysis

Western blot analysis was carried out as previously described [11,44], by incubating PVDF membranes, containing electrophoresed proteins from either nuclear or cytosolic fractions, with antibodies for CREB (Cat# 9197L RRID:AB_2245415), phospho-Ser^133^ CREB (Cat# 9198L RRID:AB_2085876), p44/42 MAPK (Cat# 9102L RRID:AB_823494), phospho-p44/42 MAPK (Thr^202^/Tyr^204^) (Cat# 8201S RRID:AB_10695902), Akt (Cat# 9272 RRID:AB_329827; 1:2000), phospho-Thr^308^ Akt (Cat# 9275L RRID:AB_329829) and phospho-Ser^473^ Akt (Cat# 9271L RRID:AB_329826), (all from Cell Signalling Technology Inc, Danvers, MA) (all 1:1000); αCaMKII 1:3000 (Millipore Cat# 05–532 RRID:AB_309787), total GSK-3α and β (EMD Millipore Cat# MABS77 RRID:AB_11205766; 1:1000), phospho-Tyr^216^ GSK-3β (EMD Millipore Cat# 05–413 RRID:AB_309721; 1:1000) and phospho-Ser^9^ GSK-3β (Millipore Cat# 07–835 RRID:AB_2115334; 1:1000) (all from Millipore S.p.A., Vimodrone, Italy), phospho-Thr^286^ αCaMKII (Thermo Fisher Scientific Cat# PA1-4614 RRID:AB_2259386; 1:2000) and β-actin 1:5000 (Sigma-Aldrich Cat# A1978 RRID:AB_476692). Following incubation with peroxidase-coupled secondary antibodies, protein bands were detected by using ECL (GE Healthcare Europe GmbH, Milano, Italy). All protein bands used were within linear range of standard curves, and both total expression and phosphorylation levels were normalized for β-actin level in the same membrane. Standardization and quantitation were performed with Quantity One software (BioRad Laboratories S.r.l., Segrate, Italy).

### Statistical analysis

Two-way ANOVA was employed for the analysis of the experiments, with treatment and time from the last administration as independent factors. When appropriate, further differences were analyzed by Bonferroni post-hoc test. Significance was assumed at p < 0.05. Statistical analysis of the data was carried out using GraphPad Prism4 (GraphPad Software Inc., USA). For the sake of simplicity in the graphs were reported only the significant Bonferroni post-hoc comparisons’ p values; the complete results of the 2-way ANOVA are described in the text. Data are presented as means ± standard error (SEM), with each individual group composed of 8 samples. For graphic clarity, optical densities from experimental groups were expressed and presented as a mean percentage of the vehicle treated group.

## Abbreviations

αCaM kinase II: Calcium/calmodulin kinase type α
a.m.: Ante meridian
AGO: Agomelatine
CaM: Calcium/calmodulin kinase
CREB: CAMP-responsive element binding protein
5-HT: Serotonin
GSK-3: Glycogen synthase kinase-3
GSK-3β: Glycogen synthase kinase-3 beta
HPC: Hippocampus
MAP kinase: Mitogen activated protein
MAP kinase/Erk1/2: Mitogen activated protein kinase Erk1 and Erk2
MT_1_/MT_2_: Melatonin receptor 1/melatonin receptor 2
p.m: post meridian
PFC/FC: Prefrontal and frontal cortex
VEH: Vehicle
